# Ohio State Dental Society

**Published:** 1877-01

**Authors:** 


### OHIO STATE DENTAL SOCIETY.

The regular annual meeting of this society was held on the 5th, 6th and 7th of December. The meeting, though not largely attended, was an excellent one. There were about fifty members present, and a goodly number of visitors. There was a very warm interest exhibited in the welfare of the society; and the prophesies of harm that should come to it, so freely indulged in by some during the last year, were not realized. There are members enough who are true to the interests of the society, to carry it through any emergency that may arise. Fortunately the emergency that, according to some, was to ruin the society, did not present any thing more than a mole hill aspect. It is a matter of regret that so many men are so easily discouraged, or are not willing to contribute the product of three or four hours work to the maintenance of a noble professional organization of our state, one that has accomplished great good for the profession, and has a great work yet to accomplish. It is greatly to be desired that the society should receive the support and co-operation of every good member of the profession in the state.

The transactions of the society will be published in a few weeks, and so we do not propose to give even a synopsis of the proceedings here.

There were six applicants for examination before the Board for certificate of qualification to practice in the state, four of whom passed a satisfactory examination; their names will appear in the transactions.

We trust that an especial effort will be made during the present year for building up and strengthening the society, and that subjects for consideration will receive due attention from every member. The list of subjects will be given in the Feb. number of the REGISTER.

